# On a more accurate half-discrete Hardy–Hilbert-type inequality related to the kernel of arc tangent function

**DOI:** 10.1186/s40064-016-2901-2

**Published:** 2016-08-11

**Authors:** Qiang Chen, Bicheng Yang

**Affiliations:** 1 Department of Computer Science, Guangdong University of Education, Guangzhou, 51003 Guangdong People’s Republic of China; 2 Department of Mathematics, Guangdong University of Education, Guangzhou, 51003 Guangdong People’s Republic of China

**Keywords:** Hardy–Hilbert-type inequality, Weight function, Hermite–Hadamard’s inequality, Equivalent form, Operator, 26D15, 47A07

## Abstract

By means of weight functions and Hermite–Hadamard’s inequality, and introducing a discrete interval variable, a more accurate half-discrete Hardy–Hilbert-type inequality related to the kernel of arc tangent function and a best possible constant factor is given, which is an extension of a published result. The equivalent forms and the operator expressions are also considered.

## Background

If $$p>1,\frac{1}{p}+\frac{1}{q}=1,f(x),g(y)\ge 0,f\in L^{p}({\mathbf {R}}_{+}),g\in L^{q}({\mathbf {R}}_{+}),||f||_{p}$$$$=(\int _{0}^{\infty }f^{p}(x)dx)^{\frac{1}{p}}>0,$$$$||g||_{q}>0,$$ then we have the following Hardy–Hilbert’s integral inequality (cf. Hardy et al. 1934):

1 $$\begin{aligned} \int _{0}^{\infty }\int _{0}^{\infty }\frac{f(x)g(y)}{x+y}dxdy<\frac{\pi }{\sin (\pi /p)}||f||_{p}||g||_{q}, \end{aligned}$$

where, the constant factor $$\frac{\pi }{\sin (\pi /p)}$$ is the best possible. Assuming that $$a_{m},b_{n}\ge 0,a=\{a_{m}\}_{m=1}^{\infty }\in l^{p},$$$$b=\{b_{n}\}_{n=1}^{\infty }\in l^{q},$$$$||a||_{p}=(\sum _{m=1}^{\infty }a_{m}^{p})^{\frac{1}{p}}>0,||b||_{q}>0,$$ we have the following Hardy–Hilbert’s inequality with the same best possible constant $$\frac{\pi }{\sin (\pi /p)}$$ (cf. Hardy et al. 1934):

2 $$\begin{aligned} \sum _{m=1}^{\infty }\sum _{n=1}^{\infty }\frac{a_{m}b_{n}}{m+n}<\frac{\pi }{\sin (\pi /p)}||a||_{p}||b||_{q}. \end{aligned}$$

Inequalities (1) and (2) are important in Analysis and its applications (cf. Hardy et al. 1934; Mitrinović et al. 1991; Yang 2009a, b, 2011).

In 1998, by introducing a parameter $\lambda \in (0,1]$, Yang (1998) gave an extension of (1) with the kernel $\frac{1}{(x+y)^{\lambda }}$ for $p=q=2$. Recently, Yang (2009b) gave extensions of (1) and (2) as follows: If $\lambda _{1},\lambda _{2}\in {\mathbf {R}},\lambda _{1}+\lambda _{2}=\lambda ,k_{\lambda }(x,y)$ is a non-negative homogeneous function of degree $-\lambda ,$ with

$$\begin{aligned} k(\lambda _{1})=\int _{0}^{\infty }k_{\lambda }(t,1)t^{\lambda _{1}-1}dt\in {\mathbf {R}}_{+}, \end{aligned}$$

$$\phi (x)=x^{p(1-\lambda _{1})-1},\psi (x)=x^{q(1-\lambda _{2})-1}, \quad f(x), g(y)\ge 0,$$

$$\begin{aligned} f\in L_{p,\phi }({\mathbf {R}}_{+})=\left\{ f;||f||_{p,\phi }:=\left( \int _{0}^{\infty }\phi (x)|f(x)|^{p}dx\right) ^{\frac{1}{p}}<\infty \right\} , \end{aligned}$$

$g\in L_{q,\psi }({\mathbf {R}}_{+}),||f||_{p,\phi },||g||_{q,\psi }>0,$ then we have

3 $$\begin{aligned} \int _{0}^{\infty }\int _{0}^{\infty }k_{\lambda }(x,y)f(x)g(y)dxdy<k(\lambda _{1})||f||_{p,\phi }||g||_{q,\psi }, \end{aligned}$$

where, the constant factor $k(\lambda _{1})$ is the best possible. Moreover, if $k_{\lambda }(x,y)$ keeps finite value and $k_{\lambda }(x,y)x^{\lambda _{1}-1}(k_{\lambda }(x,y)y^{\lambda _{2}-1})$ is decreasing with respect to $x>0\;(y>0),$ then for $a_{m,}b_{n}\ge 0,$

$$\begin{aligned} a\in l_{p,\phi }=\left\{ a;||a||_{p,\phi }:=\left( \sum _{n=1}^{\infty }\phi (n)|a_{n}|^{p}\right) ^{\frac{1}{p}}<\infty \right\} , \end{aligned}$$

$$b=\{b_{n}\}_{n=1}^{\infty }\in l_{q,\psi },$$$$||a||_{p,\phi },||b||_{q,\psi }>0,$$ we have the following Hilbert-type inequality with the same best possible constant factor $$k(\lambda _{1})$$:

4 $$\begin{aligned} \sum _{m=1}^{\infty }\sum _{n=1}^{\infty }k_{\lambda }(m,n)a_{m}b_{n}<k(\lambda _{1})||a||_{p,\phi }||b||_{q,\psi }. \end{aligned}$$

On half-discrete Hilbert-type inequalities with the non-homogeneous kernels, Hardy et al. provided a few results in Theorem 351 of Hardy et al. (1934). But they did not prove that the the constant factors are the best possible. Yang (2005) gave an inequality with the kernel $\frac{1}{(1+nx)^{\lambda }}$ as follows:

5 $$\begin{aligned} \sum _{n=1}^{\infty }\int _{0}^{\infty }\frac{a_{n}f(x)}{(1+nx)^{\lambda }}dx<B\left(\frac{\lambda }{2},\frac{\lambda }{2}\right)\left( \sum _{n=1}^{\infty }n^{1-\lambda }a_{n}^{2}\int _{0}^{\infty }x^{1-\lambda }f^{2}(x)dx\right) ^{\frac{1}{2}}, \end{aligned}$$

and proved that the constant factor $B(\frac{\lambda }{2},\frac{\lambda }{2}) (\lambda >0)$ is the best possible. Zhong et al. (Zhong 2008, 2011, 2012; Li and He 2007; Azar 2008; Jin and Debnath 2010; Huang 2010, 2015; Krnić and Vuković 2012; Adiyasuren et al. 2014, 2016; He 2015) investigated a few half-discrete Hilbert-type inequalities and some other Hilbert-type inequalities. In 2014, Yang et al. published a book (Yang and Debnath 2014) for building the theory of half-discrete Hilbert-type inequalities.

In this paper, by means of weight functions and Hermite–Hadamard’s inequality, and introducing a discrete interval variable, a more accurate half-discrete Hardy–Hilbert-type inequality related to the kernel of arc tangent function and a best possible constant factor is given, which is an extension of a published result mention in Yang and Debnath (2014). The equivalent forms and the operator expressions are considered.

## An example and some lemmas

In the following, we make appointment that $$\nu _{j}>0(j\in {\mathbf {N}}),$$$$V_{n}:=\sum _{j=1}^{n}\nu _{j}$$, $$0\le \widetilde{\nu }_{n}\le \frac{\nu _{n}}{2},$$$$\widetilde{V}_{n}=V_{n}-\widetilde{\nu }_{n},$$$$\nu (t):=\nu _{n},t\in (n-\frac{1}{2},n+\frac{1}{2}](n\in {\mathbf {N}}),$$ and

$$\begin{aligned} V(y):=\int _{\frac{1}{2}}^{y}\nu (t)dt\left(y\in \left[\frac{1}{2},\infty \right)\right), \end{aligned}$$

$$p\ne 0,1,$$$$\frac{1}{p}+\frac{1}{q}=1,\delta \in \{-1,1\},$$$$f(x),a_{n}\ge 0(x\in {\mathbf {R}}_{+},n\in {\mathbf {N}}),$$$$||f||_{p,\Phi _{\delta }}=(\int _{0}^{\infty }\Phi _{\delta }(x)f^{p}(x)dx)^{\frac{1}{p}},$$$$||a||_{q,\widetilde{\Psi }}=(\sum _{n=1}^{\infty }\widetilde{\Psi }(n)b_{n}^{q})^{\frac{1}{q}},$$ where, $$\Phi _{\delta }(x):=x^{p(1-\delta \sigma )-1},$$

$$\begin{aligned} \widetilde{\Psi }(n):=\frac{\widetilde{V}_{n}^{q(1-\sigma )-1}}{\nu _{n}^{q-1}}(x\in {\mathbf {R}}_{+},n\in {\mathbf {N}}). \end{aligned}$$

### *Example 1*

For $$\rho >0,0<\sigma <\gamma \le 1,\ $$ we set

$$\begin{aligned} h(t):=\arctan \frac{\rho }{t^{\gamma }}\,\,(t\in {\mathbf {R}}_{+}). \end{aligned}$$

(i) Setting $u=\rho ^{2}t^{-2\gamma },$ we find
  6 $$\begin{aligned} k(\sigma )&:=  {} \int _{0}^{\infty }t^{\sigma -1}\arctan \frac{\rho }{t^{\gamma }}\,dt=\frac{\rho ^{\sigma /\gamma }}{2\gamma }\int _{0}^{\infty }u^{\frac{-\sigma }{2\gamma }-1}\arctan u^{\frac{1}{2}}\,du \nonumber \\ &= \frac{\rho ^{\sigma /\gamma }}{\sigma }\int _{0}^{\infty }\arctan u^{\frac{1}{2}}du^{\frac{-\sigma }{2\gamma }} \nonumber \\ &= \frac{\rho ^{\sigma /\gamma }}{\sigma }\left[ u^{\frac{-\sigma }{2\gamma }}\arctan u^{\frac{1}{2}}|_{0}^{\infty }-\frac{1}{2}\int _{0}^{\infty }\frac{u^{\frac{-\sigma }{2\gamma }-\frac{1}{2}}}{1+u}du\right] \nonumber \\ &= \frac{\rho ^{\sigma /\gamma }}{2\sigma }\int _{0}^{\infty }\frac{u^{(\frac{1}{2}-\frac{\sigma }{2\gamma })-1}}{1+u}du=\frac{\rho ^{\sigma /\gamma }\pi }{2\sigma \sin \pi (\frac{1}{2}-\frac{\sigma }{2\gamma })} \nonumber \\ &= \frac{\rho ^{\sigma /\gamma }\pi }{2\sigma \cos (\frac{\pi \sigma }{2\gamma })}. \end{aligned}$$
(ii) We obtain for $\rho>0,0<\gamma \le 1,t>0,h(t)=\arctan \frac{\rho }{t^{\gamma }}>0,$
  $$\begin{aligned} \frac{d}{dt}h(t)=\frac{-\rho \gamma }{(t^{2\gamma }+\rho ^{2})t^{1-\gamma }}<0, \quad \frac{d^{2}}{dt^{2}}h(t)>0. \end{aligned}$$
  It is evident that for $$\sigma <1,$$$$t^{\sigma -1}h(t)>0,$$
  $$\begin{aligned} \frac{d}{dt}(t^{\sigma -1}h(t))<0, \quad \frac{d^{2}}{dt^{2}}(t^{\sigma -1}h(t))>0. \end{aligned}$$
(iii) Since for $n\in \mathbf {N,}V(y)>0,V^{\prime }(y)=\nu _{n}>0,V^{\prime \prime }(y)=0(y\in (n-\frac{1}{2},n+\frac{1}{2}),$ it follows that for $c>0,$ we have
  $$ \begin{aligned}h(cV(y))V^{{\sigma  - 1}} (y) &> 0,\quad \frac{d}{{dy}}(h(cV(y))V^{{\sigma  - 1}} (y)) < 0,  \\ \frac{{d^{2} }}{{dy^{2} }}(h(cV(y))V^{{\sigma  - 1}} (y)) &> 0\quad \left( {y \in \left( {n - \frac{1}{2},n + \frac{1}{2}} \right)} \right) \end{aligned}$$

### **Lemma 1**

*If *$$g(t)>0,g^{\prime }(t)<0,g^{\prime \prime }(t)>0$$$$(t\in $$$$\mathbf {(}\frac{1}{2},\infty )),$$*satisfying*$$\int _{\frac{1}{2}}^{\infty }g(t)dt\in {\mathbf {R}}_{+},$$*then we have*

7 $$\begin{aligned} \int _{1}^{\infty }g(t)dt<\sum _{n=1}^{\infty }g(n)<\int _{\frac{1}{2}}^{\infty }g(t)dt. \end{aligned}$$

### *Proof*

For $n_{0}\in {\mathbf {N}}\backslash \{1\},$ by the assumptions and Hermite–Hadamard’s inequality, we have

8 $$\begin{aligned} \int _{n}^{n+1}g(t)dt<g(n)<\int _{n-\frac{1}{2}}^{n+\frac{1}{2}}g(t)dt(n=1,\ldots ,n_{0}). \end{aligned}$$

It follows that

$$\begin{aligned} 0<\int _{1}^{n_{0}+1}g(t)dt<\sum _{n=1}^{n_{0}}g(n)<\sum _{n=1}^{n_{0}}\int _{n-\frac{1}{2}}^{n+\frac{1}{2}}g(t)dt=\int _{\frac{1}{2}}^{n_{0}+\frac{1}{2}}g(t)dt<\infty . \end{aligned}$$

In the same way, we still have

$$\begin{aligned} 0<\int _{n_{0}+1}^{\infty }g(t)dt\le \sum _{n=n_{0}+1}^{\infty }g(n)\le \int _{n_{0}+\frac{1}{2}}^{\infty }g(t)dt<\infty . \end{aligned}$$

Hence, adding these two inequalities, we have (7). $\square$

### **Lemma 2**

*If *$\rho >0,0<\sigma <\gamma \le 1,$*define the following weight coefficients:*

9 $$\begin{aligned} \omega _{\delta }(\sigma ,x) &:= \sum _{n=1}^{\infty }\frac{x^{\delta \sigma }\nu _{n}}{\widetilde{V}_{n}^{1-\sigma }}\arctan \frac{\rho }{(x^{\delta }\widetilde{V}_{n})^{\gamma }}, \quad x\in {\mathbf {R}}_{+}, \end{aligned}$$

10 $$\begin{aligned} \varpi _{\delta }(\sigma ,n) &:= \int _{0}^{\infty }\frac{\widetilde{V}_{n}^{\sigma }}{x^{1-\delta \sigma }}\arctan \frac{\rho }{(x^{\delta }\widetilde{V}_{n})^{\gamma }}dx, \quad n\in {\mathbf {N}}. \end{aligned}$$

*We have*

11 $$\begin{aligned} \omega _{\delta }(\sigma ,x)&<  {} k(\sigma )(x\in {\mathbf {R}}_{+}), \end{aligned}$$

12 $$\begin{aligned} \varpi _{\delta }(\sigma ,n) & =  k(\sigma )(n\in {\mathbf {N}}), \end{aligned}$$

*where, *$k(\sigma )$*is indicated by* (6).

### *Proof*

 Since

13 $$\begin{aligned} V(n) &=  \int _{\frac{1}{2}}^{n+\frac{1}{2}}\nu (t)dt-\frac{\nu _{n}}{2}=V_{n}-\frac{\nu _{n}}{2} \nonumber \\ & \le \widetilde{V}_{n}\le V_{n}=V \left(n+\frac{1}{2}\right), \end{aligned}$$

and for $t\in (n-\frac{1}{2},n+\frac{1}{2}),V^{\prime }(t)=\nu _{n},$ in view of Example 1(ii)–(iii), (13), (8) and (7), we have

$$\begin{aligned}&\frac{x^{\delta \sigma }\nu _{n}}{\widetilde{V}_{n}^{1-\sigma }}\arctan \frac{\rho }{(x^{\delta }\widetilde{V}_{n})^{\gamma }}\le \frac{x^{\delta \sigma }\nu _{n}}{V^{1-\sigma }(n)}\arctan \frac{\rho }{(x^{\delta }V(n))^{\gamma }} \\&<\int _{n-\frac{1}{2}}^{n+\frac{1}{2}}\frac{x^{\delta \sigma }V^{\prime }(t)}{V^{1-\sigma }(t)}\arctan \frac{\rho }{(x^{\delta }V(t))^{\gamma }}dt\,(n\in {\mathbf {N}}), \\ \omega _{\delta }(\sigma ,x) &< \sum _{n=1}^{\infty }\int _{n-\frac{1}{2}}^{n+\frac{1}{2}}\frac{x^{\delta \sigma }V^{\prime }(t)}{V^{1-\sigma }(t)}\arctan \frac{\rho }{(x^{\delta }V(t))^{\gamma }}dt \\ &= \int _{\frac{1}{2}}^{\infty }\frac{x^{\delta \sigma }V^{\prime }(t)}{V^{1-\sigma }(t)}\arctan \frac{\rho }{(x^{\delta }V(t))^{\gamma }}dt. \end{aligned}$$

Setting $u=x^{\delta }V(t)$ in the above, by (6), we find

$$\begin{aligned} \omega _{\delta }(\sigma ,x) &< \int _{0}^{x^{\delta }V(\infty )}\frac{x^{\delta \sigma }x^{-\delta }}{(ux^{-\delta })^{1-\sigma }}\arctan \frac{\rho }{u^{\gamma }}du \\ & \le \int _{0}^{\infty }u^{\sigma -1}\arctan \frac{\rho }{u^{\gamma }}du=k(\sigma ). \end{aligned}$$

Hence, (11) follows.

Setting $u=\widetilde{V}_{n}x^{\delta }$ in (9), we find $du=\delta \widetilde{V}_{n}x^{\delta -1}dx$. If $\delta =1,$ then

$$\begin{aligned} \varpi _{1}(\sigma ,n)=\int _{0}^{\infty }\frac{\widetilde{V}_{n}^{\sigma }\widetilde{V}_{n}^{-1}}{(\widetilde{V}_{n}^{-1}u)^{1-\sigma }}\arctan \frac{\rho }{u^{\gamma }}du=\int _{0}^{\infty }u^{\sigma -1}\arctan \frac{\rho }{u^{\gamma }}du; \end{aligned}$$

if $$\delta =-1,$$ then

$$\begin{aligned} \varpi _{-1}(\sigma ,n)=-\int _{\infty }^{0}\frac{\widetilde{V}_{n}^{\sigma }\widetilde{V}_{n}}{(\widetilde{V}_{n}u^{-1})^{1+\sigma }u^{2}}\arctan \frac{\rho }{u^{\gamma }}du=\int _{0}^{\infty }u^{\sigma -1}\arctan \frac{\rho }{u^{\gamma }}du. \end{aligned}$$

In view of (6), we have (12). $\square$

### **Lemma 3**

*If*$$\rho >0,0<\sigma <\gamma \le 1,$$*there exists a*$$n_{0}\in {\mathbf {N}},$$*such that*$$\nu _{n}\ge \nu _{n+1}$$$$(n\in \mathbf {\{}n_{0},n_{0}+1,\cdots \}),$$*and*$$V_{\infty }=\infty ,$$*then,* (i) *for*$$x\in {\mathbf {R}}_{+}\mathbf {,}$$*we have*

14 $$\begin{aligned} k(\sigma )(1-\theta _{\delta }(\sigma ,x))<\omega _{\delta }(\sigma ,x), \end{aligned}$$

*where*,

15 $$\begin{aligned} \theta _{\delta }(\sigma ,x):=\frac{1}{k(\sigma )}\int _{0}^{x^{\delta }V_{n_{0}}}u^{\sigma -1}\arctan \frac{\rho }{u^{\gamma }}du=O(x^{\delta \sigma })\in (0,1); \end{aligned}$$

(ii) * for any*$b>0,$*we have*

16 $$\begin{aligned} \sum _{n=1}^{\infty }\frac{\nu _{n}}{\widetilde{V}_{n}^{1+b}}=\frac{1}{b}\left( \frac{1}{V_{n_{0}}^{b}}+bO(1)\right) . \end{aligned}$$

### *Proof*

(i) Since for $t\in (n,n+1)(n\ge n_{0}),\nu _{n}\ge \nu _{n+1}=V^{\prime }(t+\frac{1}{2}),$ by Example 1(iii) and (8), we have

$$\begin{aligned} \omega _{\delta }(\sigma ,x)& \ge \sum _{n=n_{0}}^{\infty }\frac{x^{\delta \sigma }\nu _{n}}{V_{n}^{1-\sigma }}\arctan \frac{\rho }{(x^{\delta }V_{n})^{\gamma }} \\ &= \sum _{n=n_{0}}^{\infty }\frac{x^{\delta \sigma }\nu _{n}}{V^{1-\sigma }(n+\frac{1}{2})}\arctan \frac{\rho }{(x^{\delta }V(n+\frac{1}{2}))^{\gamma }} \\ &> \sum _{n=n_{0}}^{\infty }\int _{n}^{n+1}\frac{x^{\delta \sigma }V^{\prime }(t+\frac{1}{2})}{V^{1-\sigma }(t+\frac{1}{2})}\arctan \frac{\rho }{(x^{\delta }V(t+\frac{1}{2}))^{\gamma }}dt \\ &= \int _{n_{0}}^{\infty }\frac{x^{\delta \sigma }V^{\prime }(t+\frac{1}{2})}{V^{1-\sigma }(t+\frac{1}{2})}\arctan \frac{\rho }{(x^{\delta }V(t+\frac{1}{2}))^{\gamma }}dt. \end{aligned}$$

Setting $u=x^{\delta }V(t+\frac{1}{2})$ in the above, in view of $V_{\infty }=\infty ,$ by (6), we find

$$\begin{aligned} \omega _{\delta }(\sigma ,x) & > {} \int _{x^{\delta }V(n_{0}+\frac{1}{2})}^{\infty }u^{\sigma -1}\arctan \frac{\rho }{u^{\gamma }}du \\ &= {} k(\sigma )-\int _{0}^{x^{\delta }V_{n_{0}}}u^{\sigma -1}\arctan \frac{\rho }{u^{\gamma }}du=k(\sigma )(1-\theta _{\delta }(\sigma ,x)), \\ \theta _{\delta }(\sigma ,x) & = \frac{1}{k(\sigma )}\int _{0}^{x^{\delta }V_{n_{0}}}u^{\sigma -1}\arctan \frac{\rho }{u^{\gamma }}du\in (0,1). \end{aligned}$$

Since

$$\begin{aligned} \arctan \frac{\rho }{u^{\gamma }}\le \frac{\pi }{2}(u\in (0,\infty )), \end{aligned}$$

we find

$$\begin{aligned} 0<\theta _{\delta }(\sigma ,x)\le \frac{\pi }{2k(\sigma )}\int _{0}^{x^{\delta }V_{n_{0}}}u^{\sigma -1}du=\frac{\pi (x^{\delta }V_{n_{0}})^{\sigma }}{2\sigma k(\sigma )}, \end{aligned}$$

and then (15) follows.

(ii) For $b>0,$ by (8), we find

$$\begin{aligned} \sum _{n=1}^{\infty }\frac{\nu _{n}}{\widetilde{V}_{n}^{1+b}} &\le \sum _{n=1}^{n_{0}}\frac{\nu _{n}}{\widetilde{V}_{n}^{1+b}}+\sum _{n=n_{0}+1}^{\infty }\frac{\nu _{n}}{V^{1+b}(n)} \\ & \le \sum _{n=1}^{n_{0}}\frac{\nu _{n}}{\widetilde{V}_{n}^{1+b}}+\sum _{n=n_{0}+1}^{\infty }\int _{n-\frac{1}{2}}^{n+\frac{1}{2}}\frac{V^{\prime }(t)}{V^{1+b}(t)}dt \\ & =\sum _{n=1}^{n_{0}}\frac{\nu _{n}}{\widetilde{V}_{n}^{1+b}}+\int _{n_{0}+\frac{1}{2}}^{\infty }\frac{dV(t)}{V^{1+b}(t)} \\&=  \frac{1}{b}\left( \frac{1}{V_{n_{0}}^{b}}+b\sum _{n=1}^{n_{0}}\frac{\nu _{n}}{\widetilde{V}_{n}^{1+b}}\right) ; \\ \sum _{n=1}^{\infty }\frac{\nu _{n}}{\widetilde{V}_{n}^{1+b}}& \ge \sum _{n=n_{0}}^{\infty }\frac{\nu _{n+1}}{V^{1+b}(n+\frac{1}{2})}\ge \sum _{n=n_{0}}^{\infty }\int _{n}^{n+1}\frac{V^{\prime }(t+\frac{1}{2})}{V^{1+b}(t+\frac{1}{2})}dt \\ &= \int _{n_{0}}^{\infty }\frac{dV(t+\frac{1}{2})}{V^{1+b}(t+\frac{1}{2})}=\frac{1}{bV^{b}(n_{0}+\frac{1}{2})}=\frac{1}{bV_{n_{0}}^{b}}. \end{aligned}$$

Hence we have (16). $\square$

*Note* For example, $\nu _{n}=\frac{1}{n^{\beta }}(n\in {\mathbf {N}};0\le \beta \le 1)$ satisfies the conditions of Lemma 3 (for $n_{0}=1$).

## Main results and operator expressions

### **Theorem 1**

*If*$$\rho >0,0<\sigma <\gamma \le 1,$$$$k(\sigma )$$*is indicated by* (6), *then for*$$p>1,$$$$0<||f||_{p,\Phi _{\delta }},||a||_{q,\widetilde{\Psi }}<\infty ,$$*we have the following equivalent Hardy–Hilbert-type inequalities:*

17 $$\begin{aligned} I:& =  \sum _{n=1}^{\infty }\int _{0}^{\infty }\arctan \frac{\rho }{(x^{\delta }\widetilde{V}_{n})^{\gamma }}a_{n}f(x)dx<k(\sigma )||f||_{p,\Phi _{\delta }}||a||_{q,\widetilde{\Psi }}, \end{aligned}$$

18 $$\begin{aligned} J_{1} &:= \left\{ \sum _{n=1}^{\infty }\frac{\nu _{n}}{\widetilde{V}_{n}^{1-p\sigma }}\left[ \int _{0}^{\infty }\arctan \frac{\rho }{(x^{\delta }\widetilde{V}_{n})^{\gamma }}f(x)dx\right] ^{p}\right\} ^{\frac{1}{p}} \nonumber \\& < k(\sigma )||f||_{p,\Phi _{\delta }}, \end{aligned}$$

19 $$\begin{aligned} J_{2} &:= \left\{ \int _{0}^{\infty }\frac{1}{x^{1-q\delta \sigma }}\left[ \sum _{n=1}^{\infty }\arctan \frac{\rho }{(x^{\delta }\widetilde{V}_{n})^{\gamma }}a_{n}\right] ^{q}dx\right\} ^{\frac{1}{q}} \nonumber \\&< k(\sigma )||a||_{q,\widetilde{\Psi }}. \end{aligned}$$

### *Proof*

By Hölder’s inequality with weight (cf. Kuang 2004), we have

20 $$\begin{aligned}&\left[ \int _{0}^{\infty }\arctan \frac{\rho }{(x^{\delta }\widetilde{V}_{n})^{\gamma }}f(x)dx\right] ^{p} \nonumber \\ &= \left[ \int _{0}^{\infty }\arctan \frac{\rho }{(x^{\delta }\widetilde{V}_{n})^{\gamma }}\left( \frac{x^{\frac{1-\delta \sigma }{q}}f(x)}{\widetilde{V}_{n}^{\frac{1-\sigma }{p}}}\right) \left( \frac{\widetilde{V}_{n}^{\frac{1-\sigma }{p}}}{x^{\frac{1-\delta \sigma }{q}}}\right) dx\right] ^{p} \nonumber \\&\le \int _{0}^{\infty }\arctan \frac{\rho }{(x^{\delta }\widetilde{V}_{n})^{\gamma }}\left( \frac{x^{\frac{p(1-\delta \sigma )}{q}}f^{p}(x)}{\widetilde{V}_{n}^{1-\sigma }}\right) dx \nonumber \\& \quad \times \left[ \int _{0}^{\infty }\arctan \frac{\rho }{(x^{\delta }\widetilde{V}_{n})^{\gamma }}\frac{\widetilde{V}_{n}^{(1-\sigma )(p-1)}}{x^{1-\delta \sigma }}dx\right] ^{p-1}\nonumber \\ &= \frac{(\varpi _{\delta }(\sigma ,n))^{p-1}}{\widetilde{V}_{n}^{p\sigma -1}\nu _{n}}\int _{0}^{\infty }\arctan \frac{\rho }{(x^{\delta }\widetilde{V}_{n})^{\gamma }}\frac{x^{(1-\delta \sigma )(p-1)}\nu _{n}}{\widetilde{V}_{n}^{1-\sigma }}f^{p}(x)dx. \end{aligned}$$

In view of (12) and Lebesgue term by term integration theorem (cf. Kuang 2015), we find

21 $$\begin{aligned} J_{1}& \le (k(\sigma ))^{\frac{1}{q}}\left[ \sum _{n=1}^{\infty }\int _{0}^{\infty }\arctan \frac{\rho }{(x^{\delta }\widetilde{V}_{n})^{\gamma }}\frac{x^{(1-\delta \sigma )(p-1)}\nu _{n}}{\widetilde{V}_{n}^{1-\sigma }}f^{p}(x)dx\right] ^{\frac{1}{p}} \nonumber \\&= (k(\sigma ))^{\frac{1}{q}}\left[ \int _{0}^{\infty }\sum _{n=1}^{\infty }\arctan \frac{\rho }{(x^{\delta }\widetilde{V}_{n})^{\gamma }}\frac{x^{(1-\delta \sigma )(p-1)}\nu _{n}}{\widetilde{V}_{n}^{1-\sigma }}f^{p}(x)dx\right] ^{\frac{1}{p}} \nonumber \\ &= (k(\sigma ))^{\frac{1}{q}}\left[ \int _{0}^{\infty }\omega _{\delta }(\sigma ,x)x^{p(1-\delta \sigma )-1}f^{p}(x)dx\right] ^{\frac{1}{p}}. \end{aligned}$$

Then by (11), we have (18). By Hölder’s inequality (cf. Kuang 2004), we have

22 $$\begin{aligned} I &= \sum _{n=1}^{\infty }\left[ \frac{\nu _{n}^{\frac{1}{p}}}{\widetilde{V}_{n}^{\frac{1}{p}-\sigma }}\int _{0}^{\infty }\arctan \frac{\rho }{(x^{\delta }\widetilde{V}_{n})^{\gamma }}f(x)dx\right] \left( \frac{\widetilde{V}_{n}^{\frac{1}{p}-\sigma }a_{n}}{\nu _{n}^{\frac{1}{p}}}\right) \nonumber \\ & \le J_{1}||a||_{q,\widetilde{\Psi }}. \end{aligned}$$

In view of (18), we have (17). On the other hand, assuming that (17) is valid, we set

$$\begin{aligned} a_{n}:=\frac{\nu _{n}}{\widetilde{V}_{n}^{1-p\sigma }}\left[ \int _{0}^{\infty }\arctan \frac{\rho }{(x^{\delta }\widetilde{V}_{n})^{\gamma }}f(x)dx\right] ^{p-1}, \quad n\in {\mathbf {N}}. \end{aligned}$$

Then we find $J_{1}^{p}=||a||_{q,\widetilde{\Psi }}^{q}.$ If $J_{1}=0,$ then (18) is trivially valid; if $J_{1}=\infty ,$ then (18) keeps impossible. Suppose that $0<J_{1}<\infty .$ By (17), we have

$$\begin{aligned} ||a||_{q,\widetilde{\Psi }}^{q} &= J_{1}^{p}=I<k(\sigma )||f||_{p,\Phi _{\delta }}||a||_{q,\widetilde{\Psi }}, \\ ||a||_{q,\widetilde{\Psi }}^{q-1} &= J_{1}<k(\sigma )||f||_{p,\Phi _{\delta }}, \end{aligned}$$

and then (18) follows, which is equivalent to (17).

Still by Hölder’s inequality with weight (cf. Kuang 2004), we have

23 $$\begin{aligned}&\left[ \sum _{n=1}^{\infty }\arctan \frac{\rho }{(x^{\delta }\widetilde{V}_{n})^{\gamma }}a_{n}\right] ^{q} \nonumber \\ &= \left[ \sum _{n=1}^{\infty }\arctan \frac{\rho }{(x^{\delta }\widetilde{V}_{n})^{\gamma }}\left( \frac{x^{\frac{1-\delta \sigma }{q}}\nu _{n}^{\frac{1}{p}}}{\widetilde{V}_{n}^{\frac{1-\sigma }{p}}}\right) \left( \frac{\widetilde{V}_{n}^{\frac{1-\sigma }{p}}a_{n}}{x^{\frac{1-\delta \sigma }{q}}\nu _{n}^{\frac{1}{p}}}\right) \right] ^{q}\nonumber \\& \le  \left[ \sum _{n=1}^{\infty }\arctan \frac{\rho }{(x^{\delta }\widetilde{V}_{n})^{\gamma }}\frac{x^{(1-\delta \sigma )(p-1)}\nu _{n}}{\widetilde{V}_{n}^{1-\sigma }}\right] ^{q-1} \nonumber \nonumber \\&\times \sum _{n=1}^{\infty }\arctan \frac{\rho }{(x^{\delta }\widetilde{V}_{n})^{\gamma }}\frac{\widetilde{V}_{n}^{\frac{q(1-\sigma )}{p}}}{x^{1-\delta \sigma }\nu _{n}^{q-1}}a_{n}^{q} \nonumber \nonumber \\ &= \frac{(\omega _{\delta }(\sigma ,x))^{q-1}}{x^{q\delta \sigma -1}}\sum _{n=1}^{\infty }\arctan \frac{\rho }{(x^{\delta }\widetilde{V}_{n})^{\gamma }}\frac{\widetilde{V}_{n}^{(1-\sigma )(q-1)}}{x^{1-\delta \sigma }\nu _{n}^{q-1}}a_{n}^{q}. \end{aligned}$$

Then by (11) and Lebesgue term by term integration theorem (cf. Kuang 2015), it follows that

24 $$\begin{aligned}&J_{2}<(k(\sigma ))^{\frac{1}{p}}\left\{ \int _{0}^{\infty }\sum _{n=1}^{\infty }\arctan \frac{\rho }{(x^{\delta }\widetilde{V}_{n})^{\gamma }}\frac{\widetilde{V}_{n}^{(1-\sigma )(q-1)}}{x^{1-\delta \sigma }\nu _{n}^{q-1}}a_{n}^{q}dx\right\} ^{\frac{1}{q}}\nonumber \\ & \;= (k(\sigma ))^{\frac{1}{p}}\left\{ \sum _{n=1}^{\infty }\int _{0}^{\infty }\arctan \frac{\rho }{(x^{\delta }\widetilde{V}_{n})^{\gamma }}\frac{\widetilde{V}_{n}^{(1-\sigma )(q-1)}}{x^{1-\delta \sigma }\nu _{n}^{q-1}}a_{n}^{q}dx\right\} ^{\frac{1}{q}} \nonumber \nonumber \\ & \;= (k(\sigma ))^{\frac{1}{p}}\left\{ \sum _{n=1}^{\infty }\varpi _{\delta }(\sigma ,n)\frac{\widetilde{V}_{n}^{q(1-\sigma )-1}}{\nu _{n}^{q-1}}a_{n}^{q}\right\} ^{\frac{1}{q}}. \end{aligned}$$

In view of (12), we have (19). By Hölder’s inequality (cf. Kuang 2004), we have

25 $$\begin{aligned} I &= \int _{0}^{\infty }\left( x^{\frac{1}{q}-\delta \sigma }f(x)\right) \left[ \frac{1}{x^{\frac{1}{q}-\delta \sigma }}\sum _{n=1}^{\infty }\arctan \frac{\rho }{(x^{\delta }\widetilde{V}_{n})^{\gamma }}a_{n}\right] dx \nonumber \nonumber \\& \le ||f||_{p,\Phi _{\delta }}J_{2}. \end{aligned}$$

Then by (19), we have (17). On the other hand, assuming that (19) is valid, we set

$$\begin{aligned} f(x):=\frac{1}{x^{1-q\delta \sigma }}\left[ \sum _{n=1}^{\infty }\arctan \frac{\rho }{(x^{\delta }\widetilde{V}_{n})^{\gamma }}a_{n}\right] ^{q-1}, \quad x\in {\mathbf {R}}_{+}. \end{aligned}$$

Then we find $J_{2}^{q}=||f||_{p,\Phi _{\delta }}^{p}.$ If $J_{2}=0,$ then (19) is trivially valid; if $J_{2}=\infty ,$ then (19) keeps impossible. Suppose that $0<J_{2}<\infty .$ By (17), we have

$$\begin{aligned} ||f||_{p,\Phi _{\delta }}^{p} &= J_{2}^{q}=I<k(\sigma )||f||_{p,\Phi _{\delta }}||a||_{q,\widetilde{\Psi }}, \\ ||f||_{p,\Phi _{\delta }}^{p-1} &= J_{2}<k(\sigma )||a||_{q,\widetilde{\Psi }}, \end{aligned}$$

and then (19) follows, which is equivalent to (17).

Therefore, inequalities (17), (18) and (19) are equivalent. $\square$

### **Theorem 2**

*With regards the assumptions of Theorem*1, *if there exists a*$$n_{0}\in {\mathbf {N}},$$*such that*$$\upsilon _{n}\ge \upsilon _{n+1}$$$$(n\in \mathbf {\{}n_{0},n_{0}+1,\ldots \}),$$*and*$$V_{\infty }=\infty ,$$*then the constant factor*$$k(\sigma )$$*in* (17), (18) *and* (19) *is the best possible*.

### *Proof*

For $\varepsilon \in (0,q\sigma),$ we set $\widetilde{\sigma }=\sigma -\frac{\varepsilon }{q}(<\min \{1,\gamma \}),$ and $\widetilde{f}=\widetilde{f}(x),x\in {\mathbf {R}}_{+},\widetilde{a}=\{\widetilde{a}_{n}\}_{n=1}^{\infty },$

26 $$\begin{aligned} \widetilde{f}(x) &= \left\{ \begin{array}{ll} x^{\delta (\widetilde{\sigma }+\varepsilon )-1},&\quad 0<x^{\delta }\le 1 \\ 0,&\quad x^{\delta }>0\end{array}\right. , \end{aligned}$$

27 $$\begin{aligned} \widetilde{a}_{n} &= \widetilde{V}_{n}^{\widetilde{\sigma }-1}\nu _{n}=\widetilde{V}_{n}^{\sigma -\frac{\varepsilon }{q}-1}\nu _{n}, \quad n\in {\mathbf {N}}. \end{aligned}$$

Then for $\delta =\pm 1,$ we obtain

28 $$\begin{aligned} \int _{\{x>0;0<x^{\delta }\le 1\}}\frac{1}{x^{1-\delta \varepsilon }}dx=\frac{1}{\varepsilon }. \end{aligned}$$

By (28), (16) and (14), we find

29 $$\begin{aligned} ||\widetilde{f}||_{p,\Phi _{\delta }}||\widetilde{a}||_{q,\widetilde{\Psi }} &= \left( \int _{\{x>0;0<x^{\delta }\le 1\}}\frac{dx}{x^{1-\delta \varepsilon }}\right) ^{\frac{1}{p}}\left( \sum _{n=1}^{\infty }\frac{\nu _{n}}{\widetilde{V}_{n}^{1+\varepsilon }}\right) ^{\frac{1}{q}} \nonumber \nonumber \\ &= \frac{1}{\varepsilon }\left( \frac{1}{V_{n_{0}}^{\varepsilon }}+\varepsilon O(1)\right) ^{\frac{1}{q}}, \end{aligned}$$

$$\begin{aligned} \widetilde{I} &:= \int _{0}^{\infty }\sum _{n=1}^{\infty }\arctan \frac{\rho }{(x^{\delta }\widetilde{V}_{n})^{\gamma }}\widetilde{a}_{n}\widetilde{f}(x)dx \nonumber \\ &= \int _{\{x>0;0<x^{\delta }\le 1\}}\sum _{n=1}^{\infty }\frac{\widetilde{V}_{n}^{\widetilde{\sigma }-1}\nu _{n}}{x^{1-\delta (\widetilde{\sigma }+\varepsilon )}}\arctan \frac{\rho }{(x^{\delta }\widetilde{V}_{n})^{\gamma }}dx \nonumber \\ &= \int _{\{x>0;0<x^{\delta }\le 1\}}\omega _{\delta }(\widetilde{\sigma },x)\frac{1}{x^{1-\delta \varepsilon }}dx \nonumber \\& \ge k(\widetilde{\sigma })\int _{\{x>0;0<x^{\delta }\le 1\}}(1-O(x^{\delta (\sigma -\frac{\varepsilon }{q})}))\frac{1}{x^{1-\delta \varepsilon }}dx \nonumber \\ &= k(\widetilde{\sigma })\left[ \int _{\{x>0;0<x^{\delta }\le 1\}}\frac{dx}{x^{1-\delta \varepsilon }}-\int _{\{x>0;0<x^{\delta }\le 1\}}O\left(\frac{1}{x^{1-\delta (\sigma +\frac{\varepsilon }{p})}}\right)dx\right] \nonumber \\ &= \frac{1}{\varepsilon }k \left(\sigma -\frac{\varepsilon }{q})(1-\varepsilon O_{1}(1)\right). \end{aligned}$$

If there exists a positive constant $K\le k(\sigma ),$ such that (17) is valid when replacing $k(\sigma )$ to *K*,  then in particular, we have $\varepsilon \widetilde{I}<\varepsilon K||\widetilde{f}||_{p,\Phi _{\delta }}||\widetilde{a}||_{q,\widetilde{\Psi }},$ namely,

$$\begin{aligned} k(\sigma -\frac{\varepsilon }{q})(1-\varepsilon O_{1}(1))<K\left( \frac{1}{V_{n_{0}}^{\varepsilon }}+\varepsilon O(1)\right) ^{\frac{1}{q}}. \end{aligned}$$

It follows that $k(\sigma )\le K(\varepsilon \rightarrow 0^{+}).$ Hence, $K=k(\sigma )$ is the best possible constant factor of (17).

The constant factor $k(\sigma )$ in (18) [(19)] is still the best possible. Otherwise, we would reach a contradiction by (22) [(25)] that the constant factor in (17) is not the best possible. $\square$

For $p>1,$ we find $\widetilde{\Psi }^{1-p}(n)=\frac{\nu _{n}}{\widetilde{V}_{n}^{1-p\sigma }}(n\in {\mathbf {N}}),\Phi _{\delta }^{1-q}(x)=\frac{1}{x^{1-q\delta \sigma }}(x\in {\mathbf {R}}_{+}),$ and define the following real normed spaces:

$$\begin{aligned} L_{p,\Phi _{\delta }}({\mathbf {R}}_{+}) &= \{f;f=f(x),x\in {\mathbf {R}}_{+},||f||_{p,\Phi _{\delta }}<\infty \}, \\ l_{q,\widetilde{\Psi }} &= \{a;a=\{a_{n}\}_{n=1}^{\infty },||a||_{q,\widetilde{\Psi }}<\infty \}, \\ L_{q,\Phi _{\delta }^{1-q}}({\mathbf {R}}_{+}) &= \{h;h=h(x),x\in {\mathbf {R}}_{+},||h||_{q,\Phi _{\delta }^{1-q}}<\infty \}, \\ l_{p,\widetilde{\Psi }^{1-p}} &= \{c;c=\{c_{n}\}_{n=1}^{\infty },||c||_{p,\widetilde{\Psi }^{1-p}}<\infty \}. \end{aligned}$$

Assuming that $f\in L_{p,\Phi _{\delta }}({\mathbf {R}}_{+}),$ setting

$$\begin{aligned} c=\{c_{n}\}_{n=1}^{\infty }, \quad c_{n}:=\int _{0}^{\infty }\arctan \frac{\rho }{(x^{\delta }\widetilde{V}_{n})^{\gamma }}\,f(x)dx, \quad n\in {\mathbf {N}}, \end{aligned}$$

we can rewrite (18) as $||c||_{p,\widetilde{\Psi }^{1-p}}<k(\sigma )||f||_{p,\Phi _{\delta }}<\infty ,$ namely, $c\in l_{p,\widetilde{\Psi }^{1-p}}.$

### **Definition 1**

Define a half-discrete Hardy–Hilbert-type operator $T_{1}:L_{p,\Phi _{\delta }}({\mathbf {R}}_{+})\rightarrow l_{p,\widetilde{\Psi }^{1-p}}$ as follows: For any $f\in L_{p,\Phi _{\delta }}({\mathbf {R}}_{+}),$ there exists a unique representation $T_{1}f=c\in l_{p,\widetilde{\Psi }^{1-p}}.$ Define the formal inner product of $T_{1}f$ and $a=\{a_{n}\}_{n=1}^{\infty }\in l_{q,\widetilde{\Psi }}$ as follows:

30 $$\begin{aligned} (T_{1}f,a):=\sum _{n=1}^{\infty }\left[ \int _{0}^{\infty }\arctan \frac{\rho }{(x^{\delta }\widetilde{V}_{n})^{\gamma }}f(x)dx\right] a_{n}. \end{aligned}$$

Then we can rewrite (17) and (18) as follows:

31 $$\begin{aligned} (T_{1}f,a) &< k(\sigma )||f||_{p,\Phi _{\delta }}||a||_{q,\widetilde{\Psi }}, \end{aligned}$$

32 $$\begin{aligned} ||T_{1}f||_{p,\widetilde{\Psi }^{1-p}}& < k(\sigma )||f||_{p,\Phi _{\delta }}. \end{aligned}$$

Define the norm of operator $T_{1}$ as follows:

$$\begin{aligned} ||T_{1}||:=\sup _{f(\ne \theta )\in L_{p,\Phi _{\delta }}({\mathbf {R}}_{+})}\frac{||T_{1}f||_{p,\widetilde{\Psi }^{1-p}}}{||f||_{p,\Phi _{\delta }}}. \end{aligned}$$

Then by (32), it follows that $||T_{1}||\le k(\sigma ).$ Since by Theorem 2, the constant factor in (32) is the best possible, we have

33 $$\begin{aligned} ||T_{1}||=k(\sigma )=\frac{\rho ^{\sigma /\gamma }\pi }{2 \sigma \cos \left(\frac{\pi \sigma }{2\gamma }\right)}. \end{aligned}$$

Assuming that $a=\{a_{n}\}_{n=1}^{\infty }\in l_{q,\widetilde{\Psi }},$ setting

$$\begin{aligned} h(x):=\sum _{n=1}^{\infty }\arctan \frac{\rho }{(x^{\delta }\widetilde{V}_{n})^{\gamma }}a_{n}, \quad x\in {\mathbf {R}}_{+}, \end{aligned}$$

we can rewrite (19) as $||h||_{q,\Phi _{\delta }^{1-q}}<k(\sigma )||a||_{q,\widetilde{\Psi }}<\infty ,$ namely, $h\in L_{q,\Phi _{\delta }^{1-q}}({\mathbf {R}}_{+}).$

### **Definition 2**

Define a half-discrete Hardy–Hilbert-type operator $T_{2}:l_{q,\widetilde{\Psi }}\rightarrow L_{q,\Phi _{\delta }^{1-q}}({\mathbf {R}}_{+})$ as follows: For any $a=\{a_{n}\}_{n=1}^{\infty }\in l_{q,\widetilde{\Psi }},$ there exists a unique representation $T_{2}a=h\in L_{q,\Phi _{\delta }^{1-q}}({\mathbf {R}}_{+}).$ Define the formal inner product of $T_{2}a$ and $f\in L_{p,\Phi _{\delta }}({\mathbf {R}}_{+})$ as follows:

34 $$\begin{aligned} (T_{2}a,f):=\int _{0}^{\infty }\left[ \sum _{n=1}^{\infty }\arctan \frac{\rho }{(x^{\delta }\widetilde{V}_{n})^{\gamma }}a_{n}\right] f(x)dx. \end{aligned}$$

Then we can rewrite (17) and (19) as follows:

35 $$\begin{aligned} (T_{2}a,f) &< k(\sigma )||f||_{p,\Phi _{\delta }}||a||_{q,\widetilde{\Psi }}, \end{aligned}$$

36 $$\begin{aligned} ||T_{2}a||_{q,\Phi _{\delta }^{1-q}}& < k(\sigma )||a||_{q,\widetilde{\Psi }}. \end{aligned}$$

Define the norm of operator $T_{2}$ as follows:

$$\begin{aligned} ||T_{2}||:=\sup _{a(\ne \theta )\in l_{q,\widetilde{\Psi }}}\frac{||T_{2}a||_{q,\Phi _{\delta }^{1-q}}}{||a||_{q,\widetilde{\Psi }}}. \end{aligned}$$

Then by (36), we find $||T_{2}||\le k(\sigma ).$ Since by Theorem 2, the constant factor in (36) is the best possible, we have

37 $$\begin{aligned} ||T_{2}||=k(\sigma )=\frac{\rho ^{\sigma /\gamma }\pi }{2\sigma \cos (\frac{\pi \sigma }{2\gamma })}=||T_{1}||. \end{aligned}$$

### *Remark*

(i) For $\delta =-1$ in (17), we obtain the following inequality with the homogeneous kernel of degree 0:

38 $$\begin{aligned} \sum _{n=1}^{\infty }\int _{0}^{\infty }\arctan \left( \frac{\rho x^{\gamma }}{\widetilde{V}_{n}^{\gamma }}\right) a_{n}f(x)dx<\frac{\rho ^{\sigma /\gamma }\pi }{2\sigma \cos (\frac{\pi \sigma }{2\gamma })}||f||_{p,\Phi _{-1}}||a||_{q,\widetilde{\Psi }}. \end{aligned}$$

(ii) For $\delta =1$ in (17), we obtain the following inequality with the non-homogeneous kernel:

39 $$\begin{aligned} \sum _{n=1}^{\infty }\int _{0}^{\infty }\arctan \frac{\rho }{(x\widetilde{V}_{n})^{\gamma }}a_{n}f(x)dx<\frac{\rho ^{\sigma /\gamma }\pi }{2\sigma \cos (\frac{\pi \sigma }{2\gamma })}||f||_{p,\Phi _{1}}||a||_{q,\widetilde{\Psi }}. \end{aligned}$$

(iii) For $\widetilde{\mu }_{n}=0(n\in {\mathbf {N}})$ in (17), we have the following inequality:

40 $$\begin{aligned} \sum _{n=1}^{\infty }\int _{0}^{\infty }\arctan \frac{\rho }{(x^{\delta }V_{n})^{\gamma }}a_{n}f(x)dx<\frac{\rho ^{\sigma /\gamma }\pi }{2\sigma \cos (\frac{\pi \sigma }{2\gamma })}||f||_{p,\Phi _{\delta }}||a||_{q,\Psi }, \end{aligned}$$

where, the constant factor $\frac{\rho ^{\sigma /\gamma }\pi }{2\sigma \cos (\frac{\pi \sigma }{2\gamma })}$ is still the best possible. Hence, inequality (17) is a more accurate form of (40) (for $0<\widetilde{\mu }_{n}\le \frac{\mu _{n}}{2},n\in {\mathbf {N}}$).

(iv) For $\mu _{n}=1(x\in {\mathbf {R}}_{+},n\in {\mathbf {N}}$), $\delta =-1$ in (40), we have the following inequality:

41 $$\begin{aligned}&\sum _{n=1}^{\infty }a_{n}\int _{0}^{\infty }\arctan \rho \left( \frac{x}{n}\right) ^{\gamma }f(x)dx \nonumber \\ &= \int _{0}^{\infty }f(x)\sum _{n=1}^{\infty }\arctan \rho \left( \frac{x}{n}\right) ^{\gamma }a_{n}dx \nonumber \\& < \frac{\rho ^{\sigma /\gamma }\pi }{2\sigma \cos (\frac{\pi \sigma }{2\gamma })}\left[ \int _{0}^{\infty }x^{p(1+\sigma )-1}f^{p}(x)dx\right] ^{\frac{1}{p}}\left[ \sum _{n=1}^{\infty }n^{q(1-\sigma )-1}b_{n}^{q}\right] ^{\frac{1}{q}}, \end{aligned}$$

which is a particular case of Example 3.2 in Yang and Debnath (2014) for $\lambda =0,\lambda _{1}=-\sigma ,\lambda _{2}=\sigma$ and $k_{\lambda }(x,n)=\arctan \rho \left( \frac{x}{n}\right) ^{\gamma }$.

We still can obtain some inequalities in Theorems 1–4, by using some particular parameters.

## Conclusion

By means of the technique of real analysis, weight functions and Hermite–Hadamard’s inequality, and introducing a discrete interval variable and parameters, a more accurate half-discrete Hardy–Hilbert-type inequality related to the kernel of arc tangent function and a best possible constant factor is given. The equivalent forms and the operator expressions are also considered. The method of weight functions is very important, which is the key to help us proving the main results with the best possible constant factor. The lemmas and theorems provide an extensive account of this type of inequalities.
